# Supplementary material for: Virtual Screening for Identification of Dual Inhibitors against CDK4/6 and Aromatase Enzyme
Source: Molecules. 2023 Mar 8;28(6):2490. doi: 10.3390/molecules28062490 (PMC10058413; doi:10.3390/molecules28062490)
Supplement: Supplementary file 1 [file molecules-28-02490-s001.zip › molecules-2206230-supplementary.pdf]

Supplementary Information

Identification of promising dual inhibitors of CDK4/6 and aromatase: pharmacophore-based virtual screening, molecular docking, MD simulations, and ADMET analysis

Tenzin Adon [1], Dhivya Shanmugarajan [1], Hissana Ather [2], Shaik Mohammad Asif Ansari [3], Umme Hani [4], SubbaRao V Madhunapantula [5, 6], and Honnavalli Yogish Kumar [1,*]

[1] Department of Pharmaceutical Chemistry, JSS College of Pharmacy, JSS Academy of Higher Education and Research, Mysuru-570015, Karnataka, India
[2] Department of Pharmaceutical Chemistry, College of Pharmacy, King Khalid University (KKU), Abha – 62529, Kingdom of Saudi Arabia
[3] Department of Clinical Pharmacy, College of Pharmacy, King Khalid University (KKU), Abha – 62529, Kingdom of Saudi Arabia
[4] Department of Pharmaceutics, College of Pharmacy, King Khalid University (KKU), Abha – 62529, Kingdom of Saudi Arabia
[5] Department of Biochemistry (DST-FIST Supported Department), JSS Medical College, JSS Academy of Higher Education & Research, Mysore 570015, Karnataka, India.
[6] Special Interest Group in Cancer Biology and Cancer Stem Cells (SIG-CBCSC), JSS Academy of Higher Education & Research, Mysore 570015, Karnataka, India.

* Correspondence: yogigcp@gmail.com; +919726447802

Table of Contents

1. Table S1. The validation of structure-based pharmacophore models of CDK6 (PDB ID: 5L2S)……………………….2
2. Table S2. The validation of structure-based pharmacophore models of Aromatase (PDB ID: 3S7S)………………....2
3. Table S3. The details of known CDK4/6 inhibitors……………………………………………………………..3
4. Table S4. The details of known aromatase inhibitors…………………………………………………………..3
5. Figure S1 RSMF values of alpha carbon atoms of the CDK6 (PDB ID: 5KCV) in complex with each hit candidate and RMSF values for ligand heavy atoms bound on the active site of CDK6………………………………………4
6. Figure S2 RSMF values of alpha carbon atoms of the aromatase (PDB ID: 3S7S) in complex with each hit candidate and RMSF values for ligand heavy atoms bound on the active site of aromatase………………………………………5
7. Figure S3 The plot of CDK6 (PDB ID: 5L2S) interaction with each hit candidate and ligand atom interactions with CDK6 residues at active site………………………………………………………………………………………6
8. Figure S4 The plot of aromatase (PDB ID: 3S7S) interaction with each hit candidate and ligand atom interactions with aromatase residues at active site……………………………………………………………………………..7

Table S1. The validation of structure-based pharmacophore models of CDK6 (PDB ID: 5L2S)

Validation with Known Actives/Inactives									
Pharmacophore	Total Actives	Total Inactives	True Positives	True Negatives	False Positives	False Negatives	Sensitivity	Specificity	AUC ROC
Pharmacophore_01	17	639	10	611	28	7	0.588	0.956	0.775
Pharmacophore_02	17	639	15	571	68	2	0.882	0.893	0.909
Pharmacophore_03	17	639	11	577	62	6	0.647	0.902	0.790
Pharmacophore_04	17	639	5	632	7	12	0.294	0.989	0.643
Pharmacophore_05	17	639	14	605	34	3	0.823	0.946	0.903
Pharmacophore_06	17	639	6	601	38	11	0.352	0.940	0.654
Pharmacophore_07	17	639	11	577	62	6	0.647	0.902	0.792
Pharmacophore_08	17	639	15	531	108	2	0.882	0.830	0.904
Pharmacophore_09	17	639	12	480	159	5	0.705	0.751	0.774
Pharmacophore_10	17	639	5	606	33	12	0.294	0.948	0.625

Table S2. The validation of structure-based pharmacophore models of Aromatase (PDB ID: 3S7S)

Validation with Known Actives/Inactives									
Pharmacophore	Total Actives	Total Inactives	True Positives	True Negatives	False Positives	False Negatives	Sensitivity	Specificity	AUC ROC
Pharmacophore_01	6	289	3	90	199	3	0.500	0.311	0.555
Pharmacophore_02	6	289	3	85	204	3	0.500	0.294	0.546
Pharmacophore_03	6	289	6	14	275	0	1	0.048	0.600
Pharmacophore_04	6	289	5	13	276	1	0.833	0.044	0.570
Pharmacophore_05	6	289	6	14	275	0	1	0.048	0.679
Pharmacophore_06	6	289	6	9	280	0	1	0.031	0.704
Pharmacophore_07	6	289	6	14	275	0	1	0.048	0.593
Pharmacophore_08	6	289	5	12	277	1	0.833	0.041	0.582
Pharmacophore_09	6	289	6	8	281	0	1	0.027	0.815
Pharmacophore_10	6	289	6	10	279	0	1	0.034	0.715

Table S3. The details of known CDK4/6 inhibitors

Sl.no	Name	ID	IC_{50}	
			CDK4	CDK6
1	Abemaciclib	ZINC72318121	2 nM	10 nM
2	AMG 925	ZINC144511144	3 nM	8 nM
3	AT7519	ZINC16052857	100 nM	170 nM
4	AZD-5438	ZINC40442496	21 nM	499 nM
5	CDK4/6-IN-2	ZINC1772820533	2.7 nM	16 nM
6	Cdk4/6-IN-3	CHEMBL4545456	0.3 nM (Ki)[a]	2.2 nM (Ki)
7	CDKI-73	ZINC95605236	8.18 nM	37.68 nM
8	Dalpiciclib	ZINC219210673	12.4 nM	9.9 nM
9	FN-1501	ZINC205800206	0.85 nM	0.96 nM
10	JNJ-7706621	ZINC3938688	3 nM	175 nM
11	ON123300	ZINC103269269	3.9 nM	9.82 nM
12	Palbociclib	ZINC3938686	11 nM	16 nM
13	R547	ZINC13983251	1 nM (Ki)	4 nM
14	Ribociclib	ZINC72316335	10 nM	39 nM
15	Riviciclib	ZINC3937395	0.063 µM	0.396 µM
16	Samuraciclib	ZINC584905775	49 µM	31 µM
17	Voruciclib	SCHEMBL3108205	3.96 nM (Ki)	2.92 nM (Ki)

[a]Inhibitory constant. *Note.* Data are from https://www.medchemexpress.com/

Table S4. The details of known aromatase inhibitors

Sl. no	Name	ID	IC_{50} (aromatase)
1	Anastrozole	ZINC941	15 nM
2	Fadrozole hydrochloride	ZINC6398	6.4 nM
3	Exemestane (FCE 24304)	ZINC3973334	30 nM
4	Alpha-Naphthoflavone	ZINC38933	0.5 µM
5	Letrozole (CGS 20267)	ZINC3778874	11.5 nM
6	Androsta-1,4,6-triene-3,17-dione	ZINC4074121	0.18 µM (Ki)[a]

[a]Inhibitory constant. *Note.* Data are from https://www.medchemexpress.com/

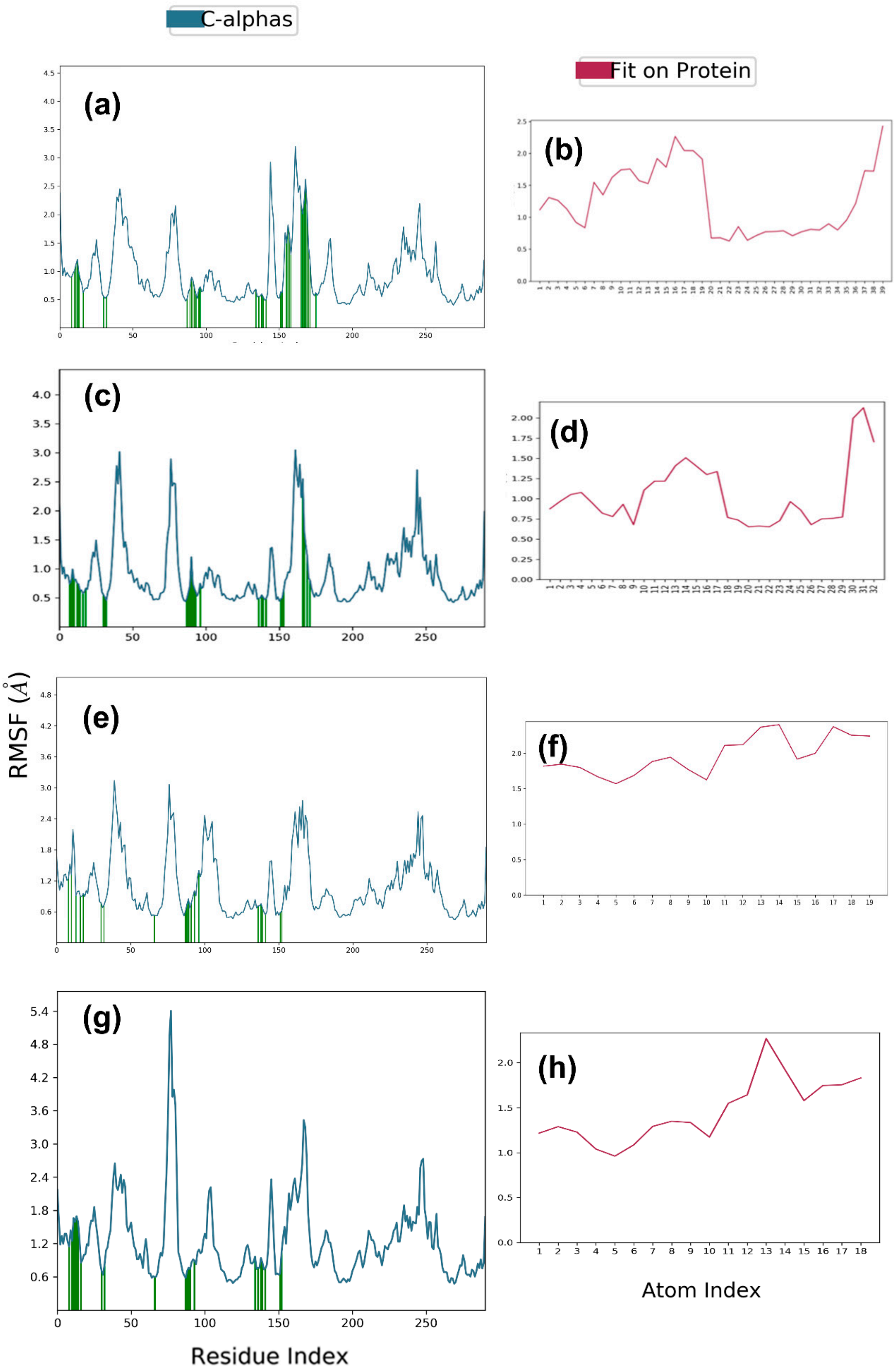

Figure S1. RSMF values of alpha carbon atoms of the CDK6 (PDB ID: 5KCV) in complex with each hit candidate and RMSF values for ligand heavy atoms bound on the active site of CDK6; (A-B) CDK6-Hit1, (C-D) CDK6-Hit2, (E-F) CDK6-Hit3, (G-H) CDK6-Hit4 calculated from the respective MD trajectories obtained from 50 ns interval.

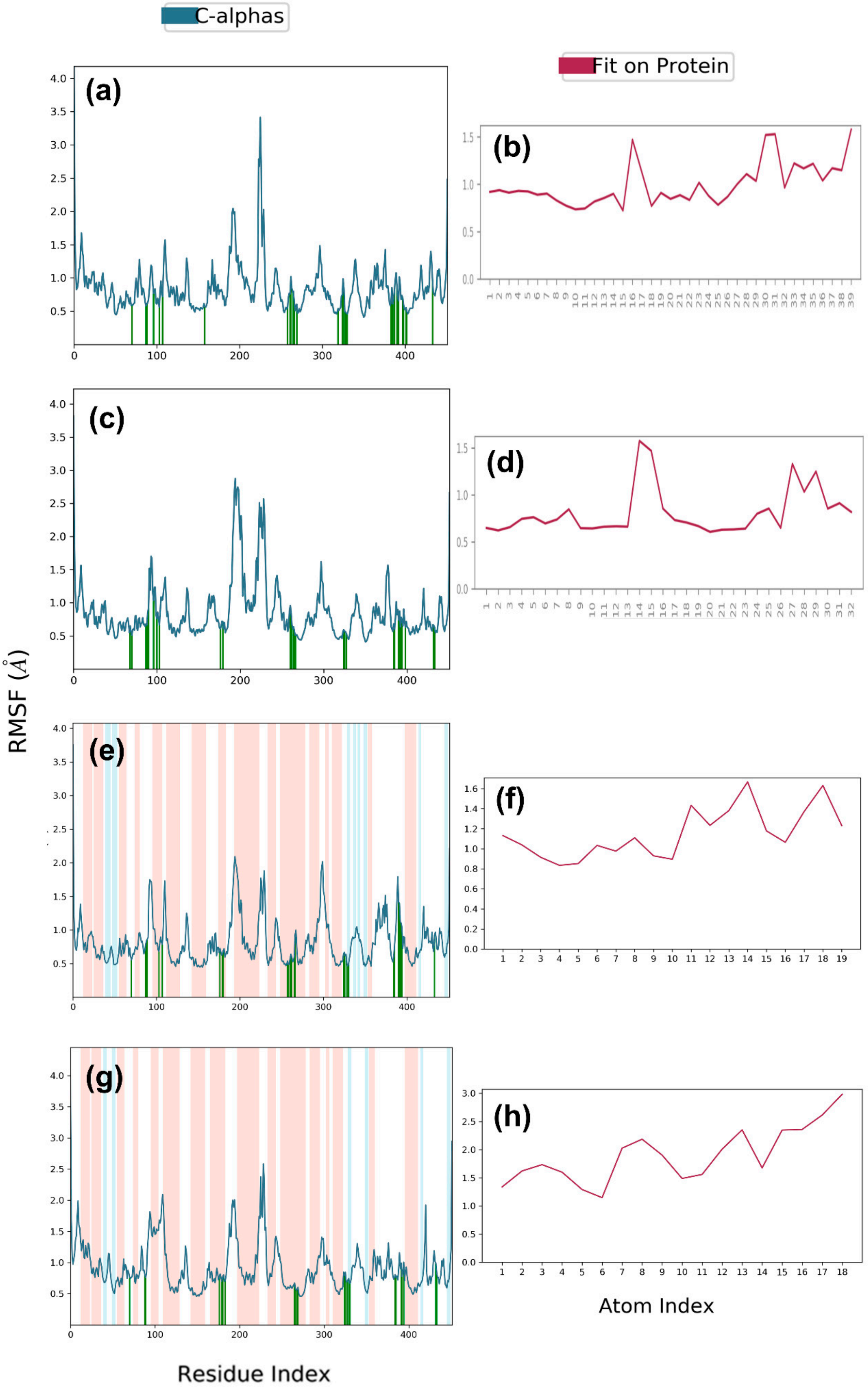

Figure S2. RSMF values of alpha carbon atoms of the aromatase (PDB ID: 3S7S) in complex with each hit candidate and RMSF values for ligand heavy atoms bound on the active site of aromatase; (A-B) aromatase-Hit 1, (C-D) aromatase-Hit 2, (E-F) aromatase-Hit 3, (G-H) aromatase-Hit 4 calculated from the respective MD trajectories obtained from 50 ns interval.

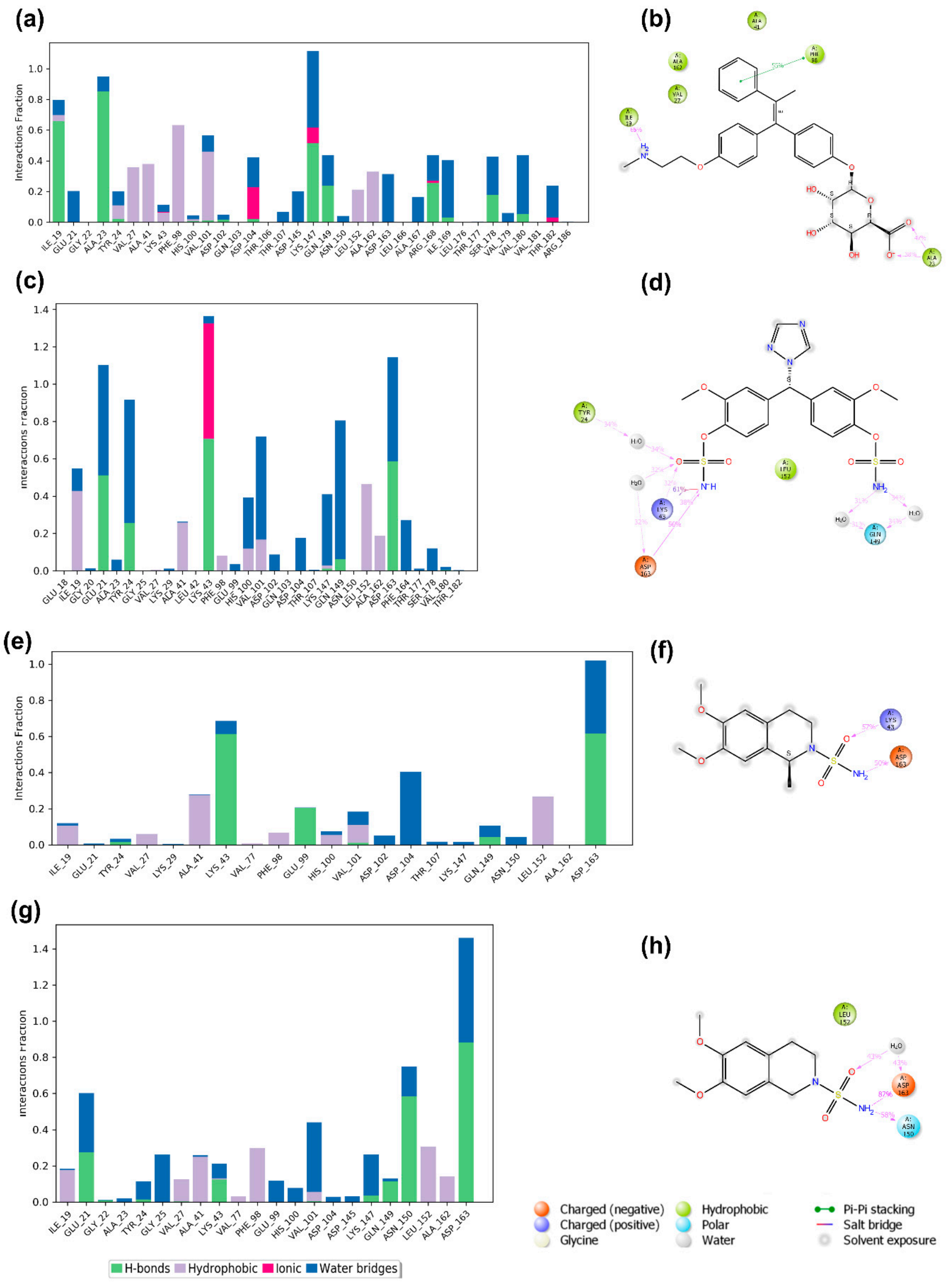

Figure S3. The plot of CDK6 (PDB ID: 5L2S) interaction with each hit candidate and ligand atom interactions with CDK6 residues at active site; (A-B) CDK6-Hit1, (C-D) CDK6-Hit 2, (E-F) CDK6-Hit 3, (G-H) CDK6-Hit 4 in the selected MD trajectories obtained from 50 ns interval. The interaction that occurs more than 30% of the simulation time is shown and more than 1.0 value of interaction fractions are plausible as some residue may take multiple contacts of the same subtype.

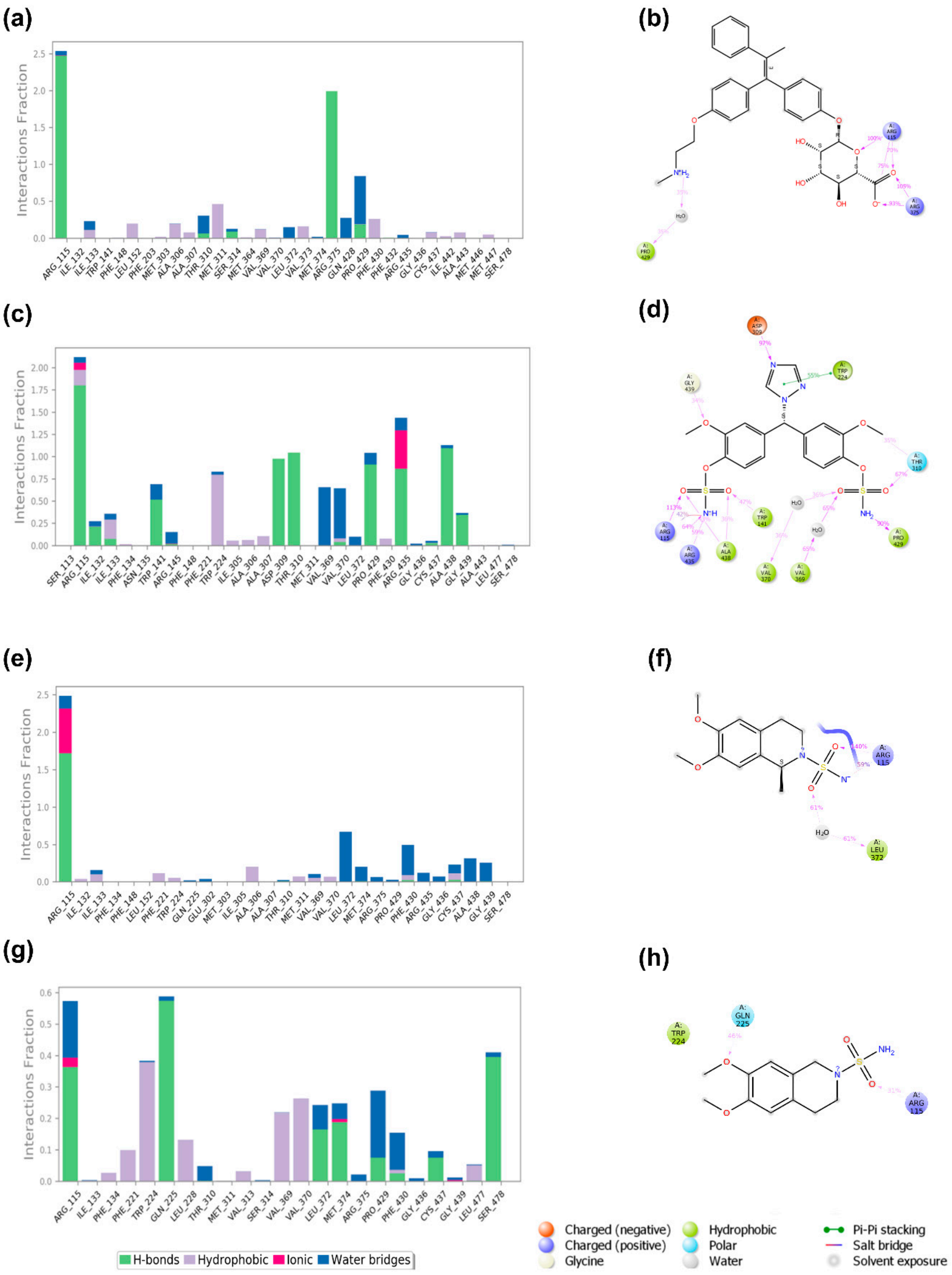

Figure S4. The plot of aromatase (PDB ID: 3S7S) interaction with each hit candidate and ligand atom interactions with aromatase residues at active site; (A-B) aromatase-Hit 1, (C-D) aromatase-Hit 2, (E-F) aromatase-Hit 3, (G-H) aromatase-Hit 4 in the selected MD trajectories obtained from 50 ns interval. The interaction that occurs more than 30% of the simulation time is shown and more than 1.0 value of interaction fractions are plausible as some residue may take multiple contacts of the same subtype.
